# Prolonged migraine aura: new insights from a prospective diary-aided study

**DOI:** 10.1186/s10194-018-0910-y

**Published:** 2018-08-31

**Authors:** Michele Viana, Grazia Sances, Mattias Linde, Giuseppe Nappi, Farihah Khaliq, Peter J. Goadsby, Cristina Tassorelli

**Affiliations:** 1 Headache Science Center, IRCCS Mondino Foundation, Pavia, Italy; 2 Department of Neuroscience, Norwegian University of Science and Technology, Trondheim, Norway; 3 Norwegian Advisory Unit on Headaches, St. Olavs University Hospital, Trondheim, Norway; 4 Headache Group - NIHR-Wellcome Trust King’s Clinical Research Facility, King’s College, London, UK; 5 Dept of Brain and Behavioural Sciences, University of Pavia, Pavia, Italy

**Keywords:** Prolonged Aura, Migraine with aura, Duration, Features, Headache

## Abstract

**Background:**

There is limited literature on prolonged aura (PA - defined as an aura including at least one symptom for > 1 h and < 7d), and there are no prospective studies.

The aim of this study is to characterize prospectively the phenotype and prevalence of PA.

**Findings:**

Two hundred and twenty-four patients suffering from migraine with aura were recruited from the Headache Centers of Pavia and Trondheim. Patients prospectively described, on an ad hoc diary, each aura symptom (AS), the duration of AS and headache, and headache features. Seventy-two patients recorded three consecutive auras in their diaries. 19 (26.4%) of patients suffered at least one PA. Out of 216 recorded auras, 38 (17.6%) were PAs. We compared PAs with non-PAs with respect to 20 features; PAs were characterized by a higher number of non-visual symptoms (non-VS) (*p* < 0.001). No other differences were found. We obtained similar results when we compared auras with at least one symptom with a duration of > 2 h (*n* = 23) or > 4 h (*n* = 14) with the the others (*n* = 193 and *n* = 202 respectively).

**Conclusion:**

PAs are quite common. They do not differ from the other auras (even when their duration extends to 2 and/or 4 h) with the exception of a higher number of non-VS.

## Introduction

Worldwide migraine is the third most common disorder [1] and around 30% of sufferers experience migraine auras [2]. Aura comprises completely reversible visual, sensory, or language symptoms (occurring respectively in 98, 36 and 10% of auras) [3].

All of the first three editions of the International Classification of Headache Disorders [4–6] (ICHD) have considered the individual symptoms of aura to be typical if the duration is more than five and less than 60 min. However these symptoms can last longer in a rare subtype of migraine with aura, namely hemiplegic migraine, which is not discussed in this paper.

The first version of the ICHD included migraine with prolonged aura (PA) and defined it as migraine with one or more aura symptoms lasting more than 60 min and less than a week, occurring in the presence of normal neuroimaging findings [5]. However the subsequent two versions of the ICHD removed PA from the classification [5, 6].

Currently a prolonged non-hemiplegic migraine with aura (NHMA) is classified as ‘persistent aura without infarction’ if the duration is equal or longer than 7 days. Those lasting more than 60 min and less than 7 days are classified as ‘probable migraine with aura (prolonged aura)’.

The term “probable” used in such classification indicates suspicion as to whether the symptom is migraine aura and from our clinical experience we feel it does not help to categorise auras of a longer duration. We feel that a detailed description of the phenotype of the attacks it is important for achieving a more evidence-based nosographic framing of migraine with aura.

So far there are no systematic studies assessing prospectively in adults i) the prevalence of PA and ii) the clinical characteristics of PA. In the literature we could find only a retrospective study conducted in pediatric patients [7], one case series reported in the abstract form to a congress [8] and few case reports [9, 10].

We present a prospective study focusing on the frequency of occurrence and characteristics of PA. This study forms part of a larger research dedicated to the evaluation of the temporal and qualitative aspects of migraine with aura, whose results have been partly published in 2 previous reports [3, 11]. Preliminary results was presented during the International Headache Congress, Vancouver, Canada, 2017 [12].

## Methods

We enrolled 224 successive patients in the headache centers of Pavia and Trondheim (198 and 26 respectively) who suffered from migraine with aura. The recruitment period was between October 2012 and July 2014, with the completion of follow-up in May 2015. All patients provided signed informed consent and ethical approval was sought from local ethics committees (“C. Mondino” National Neurological Institute, Pavia, Italy and REC-Central, Trondheim, Norway).

Inclusion criteria were: patients suffering from migraine with aura for at least 1 year which met the ICHD-2 criteria for 1.2.1 [G43.10] typical aura with migraine headache,1.2.2 [G43.10], typical aura with non-migraine headache, 1.2.3 [G43.104] typical aura without headache, excluding point 3 of C criteria (“each individual aura symptom lasts 5-60 minutes”) and where only one of point C1 or C2 had to be verified to fulfill C criteria; ii) age between 16 and 65 years.

Exclusion criteria were: i) hemiplegic migraine; ii) brainstem aura; iii) pregnancy; iv) variation of the characteristics of aura and/or headache in the last 6 months: v) patients with > 2 vascular risk factors; vi) history of myocardial infarction and/or transitory ischemic attack (TIA) and/or stroke and/or others thrombophilic disturbances; vii) patients with episodes that are not clearly differentiated from other disturbances (TIA, seizures).

Each patient was diagnosed with migraine with aura by a neurologist of the headache center, who then confirmed the patient met the inclusion and exclusion criteria before being included in the study. Patients were asked to prospectively record the characteristics of three consecutive attacks in an ad hoc aura diary. The diary allowed the patients to - describe each visual (VS), sensory (SS) and dysphasic (DS) aura symptom in their own words, highlight the main characteristics of their migraine and record the duration of the aura symptoms and headache. Follow up visits were arranged with a neurologist for patients who had completed three recordings and the content of their diaries was discussed, specifically to verify if the described symptoms i) were typical of previous auras experienced by the patient ii) were not premonitory symptoms (i.e. photophobia, difficulty with concentration/speech).

During the analysis of the data, all reported visual phenomena were grouped into the elementary visual symptoms as described by Queiroz and colleagues [13] including ‘visual snow’ (small black/grey dots on a light background and grey/white dots on a dark background) [14] and ‘deformed images’ (altered object shapes). In cases where two symptoms occurred simultaneously, the first symptom (FS) was considered to be the one that ended first.

### Statistical analysis

Continuous variables are reported using medians and the interquartile range (IQR) whereas categorical variables are reported using percentages or means and the standard deviation.

In order to statistically compare groups a chi-square test was used for categorical variables and the Kruskall-Wallis test was employed for continuous variables.

As this was an investigative study, nominally significant statistics (*p* value < 0.05) were reported. To avoid accidental findings as multiple tests were carried out making 21 comparisons in total, adjusted *p*-values based on the Bonferroni correction were used. A lower level of significance was also used *p* < 0.00238 (*p* = 0.05/21). The statistical analysis was performed using MedCalc Version 13.3.3 (MedCalc Software, Mariakerke, Belgium) software.

## Findings

### Patients

We recruited 224 patients, 72 of which recorded three consecutive auras (64 from Pavia and eight from Trondheim), giving a total number of 216 auras, which were included in the analysis. From the remaining participants 37 decided to drop out of the study and 115 were not included as they did not manage to record 3 auras. The features of the auras experienced by the 72 participants who were analysed are reported in Table 1.

**Table 1:** Characteristics of patients (*n* = 72). Data is presented as means ± SD (range) for continuous data and as n (% of column) for categorical variables

Variable	Patients (*n* = 72)
Female	58 (80%)
Age (years)	41 ± 14 (18–65)
Age at onset of migraine with aura (years)	22 ± 20 (6–60)
Frequency of migraine with aura (attacks/year)	21 ± 12 (2–130)
Headache	
on 3/3 attacks	63 (88)
on 1/3 or 2/3 attacks	6 (8)
on 0 attacks	3 (4)
Co-occurrence of migraine without aura	54 (75)
Age at onset of migraine without aura (years)	16 ± 14 (7–45)
Co-occurrence of tension type headache	10 (14)
Family history of migraine with aura^a^	15 (21)
Use of preventive drugs during the study period	26 (36)
Betablockers	5
Calcium channel blockers	5
Tricyclic acids	6
Antiepileptic drugs	8
Pizotifen	1
Antiepileptic drugs + betablockers	1

^a^First and second-degree relatives were considered

Out of these 72 participants 19 (26.4%) experienced at least one PA. Of these 19 patients, nine experienced three PAs, whereas ten patients experienced one or two PAs out of the three auras.

### Features of auras

The features of PA and non-PA are reported in Table 2.

**Table 2:** Characteristics of PA (auras with at least 1 symptoms lasting > 1 h) and NON-PA

	Prolonged auras (PA)	NON-PA	Sig.
Number	38	178	
Visual symptoms (VS)	37 (97)	175 (98)	0.69
DVP	19 (50)	77 (43)	0.44
Positive	23 (60)	119 (67)	0.45
Negative	12 (31)	70 (39)	0.37
Number of elementary visual disturbance^ per aura	1.97 (1.05)	1.94 (0.93)	0.83
Sensory symptoms (SS)	26 (68)	49 (27)	**< 0.0001**
Dysphasic symptoms (DS)	12 (31)	10 (7)	**< 0.0001**
Number of symptoms (VS, SS, DS)/aura			**< 0.0001**
1	10 (26)	127 (71)	
2	19 (50)	46 (26)	
3	9 (23)	5 (3)	
Time relation between aura symptoms in one aura			0.28
B^a^ starts simultaneously with A^a^	11 (42)	14 (27)	
B starts during A	11 (42)	18 (37)	
B starts when A ceased	2 (8)	5 (10)	
B starts after an interval of time after A has ceased	2 (8)	12 (24)	
Headache	38 (100)	159 (83)	0.10
Started before/together with Aura (*n* = 157)	7 (22)	27 (21)	0.88
Intensity (*n* = 192)	2.3 (0.8)	2.2 (0.8)	0.59
Unilateral pain (*n* = 192)	27 (75)	100 (64)	0.21
Throbbing pain (*n* = 187)	17 (47)	71 (47)	0.98
Pain aggravated by physical activity (*n* = 189)	27 (73)	98 (65)	0.32
AS: nausea (*n* = 192)	23 (62)	101 (65)	0.73
AS: vomiting (*n* = 189)	8 (23)	25 (16)	0.35
AS: photophobia (*n* = 192)	29 (78)	126 (81)	0.68
AS: phonophobia (*n* = 191)	23 (62)	99 (64)	0.81
AS: osmophobia (*n* = 189)	6 (43)	53 (34)	0.31

Data are presented as means (SD) for continuous data and as n (% of column) for categorical variables. ^In the analysis, the authors dissected every visual into elementary disturbance as reported by Viana et al. (2016) [12]

^a^A and B can be referred respectively to 1st and 2nd aura symptoms or 2nd and 3rd aura symptoms (67 auras had at least 2 symptoms, 14 auras had 3 symptoms). When two symptoms started simultaneously, we designated the first completing symptom as A

Abbreviations: *DVP* disturbances of visual perception (i.e. blurred/foggy vision, ‘like looking through heat waves or water’, deformed images), *AS* Associated symptom

Note: In bold, data referred to variables that reached a statistical significance (*p* < 0.05) in such analysis

With respect to temporal aspects, the median duration was 135 min (IQR 630) for VSs, 180 min (IQR 390) for SSs, 70 min (IQR 35) for DSs. One PA had 3 prolonged symptoms (> 1 h), six PAs had two prolonged symptoms (VS + SS = 5, VS + DS =1), 31 PAs had only one prolonged symptom (VS = 19, SS = 11, DS = 1). When comparing PAs with the other auras (*n* = 178) we found PAs were characterized by a higher total number of symptoms (*p* < 0.001), a higher frequency of SSs (*p* < 0.001) and a higher frequency of DSs (*p* < 0.001). No other differences were found.

We then carried out two further similar analyses, firstly comparing auras with a minimum of one symptom lasting for > 2 h (PA > 2, *n* = 23) with the others (*n* = 193). The only differences found was a higher frequency of SSs and a higher number of aura symptoms in PA > 2 (*p* = 0.001 and *p* = 0.005, respectively). In the second comparison auras with a minimum of one symptom lasting for > 4 h (PA > 4, *n* = 14) were compared with the others (*n* = 202). The only difference was a higher number of aura symptoms in PA > 4 (*p* = 0.043).

## Discussion

This study prospectively analysed a significant number of migraine auras recorded with the use of an ad hoc diary, allowing us to define the features and the frequency of occurrence of PAs. Our findings show that phenotypically PAs are similar to non-PAs and are fairly common with 17% of all auras being PA and with 26% of patients experiencing at least one. The only differences found between PAs and other auras was a higher number of non-visual symptoms.

This can be expected if we consider the pathophysiology of auras and recognize that aura symptoms with a longer duration are likely to be related to a cortical spreading depression (CSD) proceeding across a longer path on the respective brain area. This type of CSD is more likely to spread and involve adjacent brain areas, therefore producing additional non-VSs to the PA. The similarity in phenotypes of PAs and the other auras continues to be significant when the maximum duration is increased to 2 and/or 4 h.

A key limitation of this research is that the study population was recruited from tertiary headache centers and therefore it can be argued that the population is not representative as they are likely to be more difficult cases. However as all patients were seen by a headache specialist the accuracy of the results are not refuted. The use of a patient diary in a population-based study is always challenging and the level of detail to be included in the clinical evaluation poses an issue. Nevertheless our research is the first specifically evaluating the occurrence of PA conducted on an adult population.

**Fig. 1:**
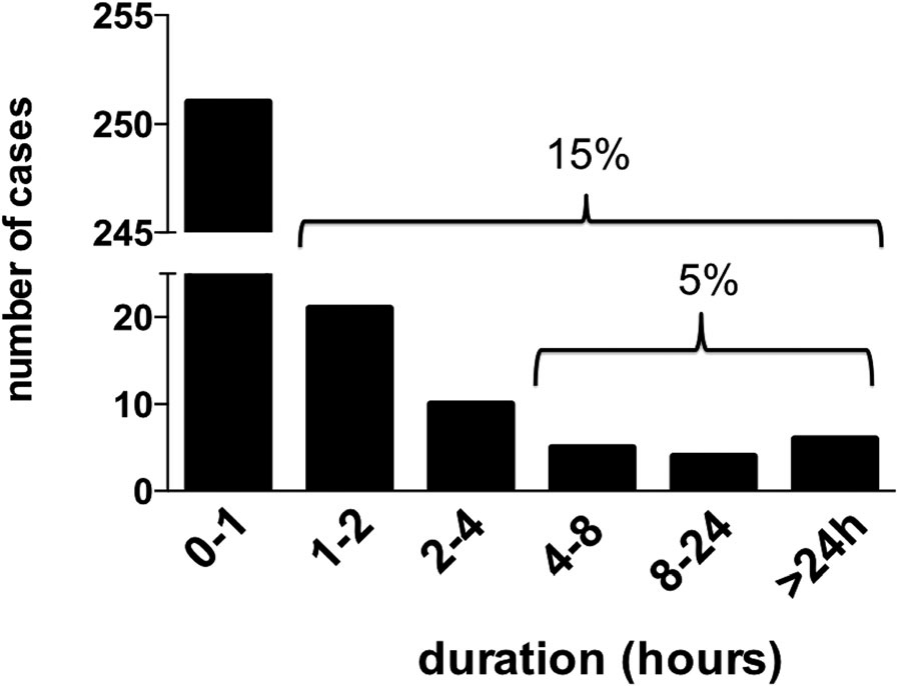
Distribution of duration of all aura symptoms (*n*=297). Raw data reported in Viana et al. [3]

Our findings indicate the need to reconsider the use of the term “prolonged aura” and the duration of aura symptoms that should be classed as a typical or prolonged auras. Generally speaking, an event is considered typical if it describes 95% of a given sample. If we look at the distribution of the duration of all the aura symptoms recorded in our study (see figure - raw data already described in a previous study [3]), 95% of aura symptoms last less than 4 h, while only 5% exceed this Fig. 1. Therefore, from a purely statistical point of view, it would be correct to raise the time limit of aura symptoms up to 4 h to be considered a PA.

If the reason to be more cautious is to avoid misdiagnosing an aura with other neurological disorders (such as cerebrovascular diseases), then one could argue that adopting a time limit of 2 h would be conservative enough. With these figures in mind, it would then be useful to discuss whether it is worth reintroducing the entity “migraine with prolonged aura” in the classification Fig. 1.

## Conclusions

Prolonged auras are quite common, being 17% of all auras, occurring at least once in 26% of patients. They are phenotypically similar to the other auras. Our findings indicate the need to reconsider the use of the term “prolonged aura” and the duration of aura symptoms that should be classed as a typical or prolonged auras.

## Abbreviations

AS: Aura symptom
DS: Dysphasic symptom
DVP: Disturbances of visual perception
ICHD: International Classification of Headache Disorders
IQR: Interquartile range
NHMA: Non-hemiplegic migraine with aura
PA: Prolonged aura
SS: Sensory symptom
VS: Visual symptom
